# The Voice of Bats: How Greater Mouse-eared Bats Recognize Individuals Based on Their Echolocation Calls

**DOI:** 10.1371/journal.pcbi.1000400

**Published:** 2009-06-05

**Authors:** Yossi Yovel, Mariana Laura Melcon, Matthias O. Franz, Annette Denzinger, Hans-Ulrich Schnitzler

**Affiliations:** 1 Animal Physiology, Institute for Neurobiology, University of Tuebingen, Tuebingen, Germany; 2 University of Applied Sciences, Konstanz, Germany; University College London, United Kingdom

## Abstract

Echolocating bats use the echoes from their echolocation calls to perceive their surroundings. The ability to use these continuously emitted calls, whose main function is not communication, for recognition of individual conspecifics might facilitate many of the social behaviours observed in bats. Several studies of individual-specific information in echolocation calls found some evidence for its existence but did not quantify or explain it. We used a direct paradigm to show that greater mouse-eared bats (*Myotis myotis*) can easily discriminate between individuals based on their echolocation calls and that they can generalize their knowledge to discriminate new individuals that they were not trained to recognize. We conclude that, despite their high variability, broadband bat-echolocation calls contain individual-specific information that is sufficient for recognition. An analysis of the call spectra showed that formant-related features are suitable cues for individual recognition. As a model for the bat's decision strategy, we trained nonlinear statistical classifiers to reproduce the behaviour of the bats, namely to repeat correct and incorrect decisions of the bats. The comparison of the bats with the model strongly implies that the bats are using a prototype classification approach: they learn the average call characteristics of individuals and use them as a reference for classification.

## Author Summary

Animals must recognize each other in order to engage in social behaviour. Vocal communication signals could be helpful for recognizing individuals, especially in nocturnal organisms such as bats. Echolocating bats continuously emit special vocalizations, known as echolocation calls, and perceive their surroundings by analyzing the returning echoes. In this work we show that bats can use these vocalizations for the recognition of individuals, despite the fact that their main function is not communication. We used a statistical approach to analyze how the bats could do so. We created a computer model that reproduces the recognition behaviour of the bats. Our model suggests that the bats learn the average calls of other individuals and recognize individuals by comparing their calls with the learnt average representations.

## Introduction

Voice is defined as the entirety of all acoustic signals produced by the vocal organs of an organism and its ability to produce them. Vocalizations are mostly used for communication. They can contain information about identity, gender, maturity, health, behavioural context, etc [1]–[3]. Specific properties of the sound production and articulation apparatus are responsible for the individual-specific spectral properties of vocalizations. The human voice, for instance, reveals the identity of individuals and lately it has been shown that other animals can also recognize individuals according to their social vocalizations [4]–[10]. Social vocalizations constitute an important part of the vocal repertoire of bats. These vocalizations have been characterized for many species and contexts and were shown to contain individual signatures [11]–[23]. In addition to social vocalizations, microchiropteran bats constantly emit echolocation calls and use the returning echoes to perceive their surroundings [24]. These echolocation calls are tonal signals that exhibit a structured change in frequency over time that is normally less variable than that of the social vocalizations. The ability to recognize individuals based on echolocation calls might explain many of the social behaviours observed in bats [e.g., 16]. Several studies tried to find individual-specific cues in bat echolocation calls [2], [25]–[28]. Recently, the response of bats to the echolocation calls of different individuals has been tested and the results suggested that they could recognize individuals according to their echolocation calls [29].

The echolocation calls of the greater mouse-eared bats (*Myotis myotis*) used in this study are ∼3 ms long frequency-modulated (FM) down-sweeps ranging from ∼100 kHz to ∼30 kHz. The exact spectral-temporal structure of the calls changed depending on the task. We hypothesize that, despite this variability, the echolocation signals might contain individual-specific characteristics, generated by the bats' vocal apparatus, which are sufficient for individual recognition. We first tested whether bats can distinguish between individuals according to their echolocation calls using the most direct approach used until today: training greater mouse-eared bats to classify echolocation calls of other individuals played back to them in a two alternative forced choice (2-AFC) experiment. After showing that the bats can clearly recognize their conspecifics, we used a statistical approach, new in this field, to train statistical classifiers to reproduce the bats' behaviour, namely to make similar correct and incorrect decisions as the bats. Our approach offers two main advantages in comparison to former unsuccessful attempts to statistically identify individual bats according to their echolocation calls [30]. First, our method is almost unlimited in the number of parameters that can be fed into it. This enabled us to use the raw representations of the calls and not to limit ourselves to a set of parameters as was always the case before. Second, we used a large data set containing ca. 800 calls per bat. Such a large data set enables us to create a good model of the individual's call despite its large variability. We used the statistical classifier as a model of the bat's underlying decision process to show how classification is statistically possible and to understand how the bats might be able to recognize other individuals.

## Results

### Echolocation calls

**Figure 1:**
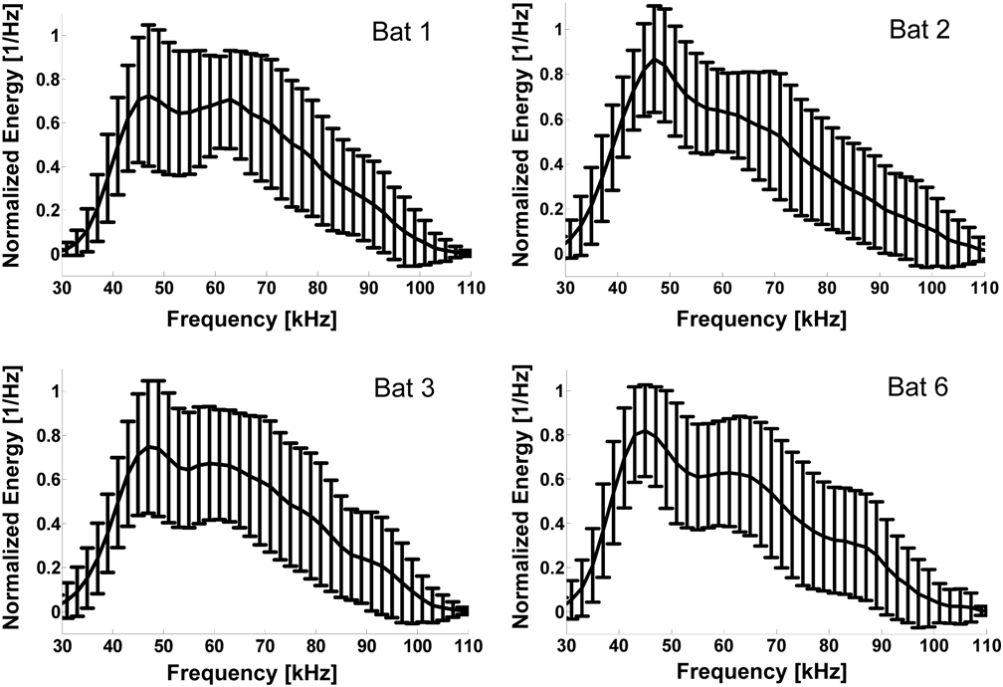
Normalized spectra (means and SD) of four of the bats used in the experiments. Each spectrum was normalized to have a maximum of 1. Note the overall similar shape and the high variability.

All bats emitted calls typical for flying in confined spaces with a very characteristic spectral-temporal structure. Despite this repeating pattern, the spectral content of the calls varied largely among individuals for both behavioral and technical reasons (see Materials and Methods and Figure 1). There was also some intra-individual variability of the sweep rate (Table 1) depicting the differences in the time structure of the calls. Finally, it is worth emphasizing that the SNR of the calls varied dramatically (Table 1) as a result of the varying distance from the microphone.

**Table 1:** Basic Call Parameters.

	Bat 1	Bat 2	Bat 3	Bat 5	Bat 6
Call Duration (ms)	2.5±0.2	2.6±0.3	2.6±0.2	2.5±0.3	2.7±0.3
Starting Frequency (kHz)	96±8	98±10	96±6	93±7	95±9
Terminal Frequency (kHz)	34±3	34±3	37±3	36±3	34±3
Maximum Energy Frequency (kHz)	56±11	54±11	57±11	63±12	56±13
Sweep Rate (kHz/ms)	25±4	25±4	24±4	23±3	23±4
SNR	31±36	26±32	39±40	35±40	38±41

Basic calls parameters (mean+SD) for the bats whose calls were used in the experiments. The onset and end of the calls were defined to be 25 dB lower than the maximum. SNR was calculated as the ratio between the maximum call amplitude and the maximum noise amplitude determined from the spectrogram background.

### Behavioral classification experiments

**Figure 2:**
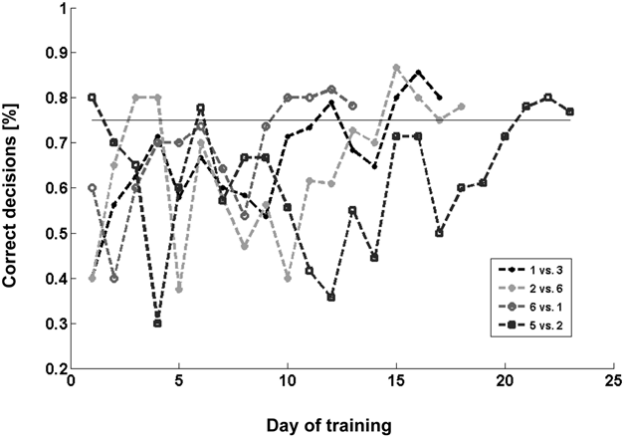
The learning curves. The correct decision percentage is presented as a function of the day of training for each bat. Training was stopped once a bat performed 75% or more of the trials for three consecutive days.

The bats required 15–24 days before they were able to stably correctly recognize the individuals in more than 75% of the trials. The learning curves (Figure 2) fluctuated between days. After training, all bats were able to recognize S+ (a single call of the bat they learned to recognize) with much higher accuracy than chance level (Table 2).

**Table 2:** Bat Performance.

Experimental task	S+ vs. S− S+ approach percentage	S+ vs. S0 S+ approach percentage	S0 vs. S− S− avoidance percentage
Bat 3 vs. bat 1	77% (125)	95% (40)	70% (40)
Bat 2 vs. bat 6	83% (96)	95% (40)	80% (40)
Bat 6 vs. bat 1	90% (126)	85% (40)	75% (40)
Bat 5 vs. bat 2	91% (122)	95% (40)	60% (40)

Overall percent of correct decisions in the test phase. Numbers in brackets depict the number of trials. The S+ and S− columns present the behavior for the controls with calls of the new bats. 95% for S+ means that the bat approached S+ in 95% of the trials when played along with S0 and 70% for S− means that the bat avoided S− in 70% of trials when played with S0.

### Test of generality

Bats were able to generalize from the learned task to recognize S+ or avoid S− (a single call of the bat that they learned to avoid) when presented with calls of new bats that were never heard during training (Table 2). Most of the bats showed both a preference for S+ and an avoidance of S−. The higher percentage of approaching S+ when presented with S0 (a single call of a bat that they did not encounter during training) can be a result of the fact that the S+ calls in these experiments were taken from the training set and thus - the bats might have already heard them during training. The lower avoidance of S− when presented with S0 could result from the fact that they were familiar to the bats and the bats were even rewarded when approaching them during the test phase.

### Machine classification

A linear classifier (Support Vector Machine – SVM) learned to classify the calls with high accuracy (correct decision rates of 81–90%). This was the case for both types of representations of the calls, i.e. the temporal-spectral spectrograms and the spectral power spectrum densities (PSD, Table 3) although in the case of the PSDs the performance was a bit lower (77–84%). This indicates that individual-specific information is abundant in the calls. The overall performance of the linear machines was similar to that of the bats.

**Table 3:** The Performance of Linear and Non-linear SVM Classifiers.

Classification task\Information used	Bat 5 vs. bat 2	Bat 6 vs. bat 1	Bat 2 vs. bat 6	Bat 1 vs. bat 3
**Linear classifier**				
**Time+Frequency**	90±11%	84±16%	81±11%	87±7%
C	10	100	1	1
Correlation with bat performance	−0.12±0.40	−0.15±0.30	−0.53±0.15	−0.15±0.08
Identical decisions	90±6% (82)	83±3% (77)	74±8% (71)	69±2% (70)
**Frequency**	79±11	77±9	84±8%	84±5%
C	0.1	50	10	1
Correlation with bat performance	−0.05±0.18	0.55±0.44	−0.05±0.12	−0.05±2%
Identical decisions	72±4% (74)	85±12% (78)	74±4% (72)	70±2% (69)
**Non-linear classifier**				
**Time+Frequency**	94±3%	91±1%	77±1%	82±3%
C, σ	10,5	20,5	50,100	1,5
Correlation with bat performance	0.45±0.05	0.15±0.08	0.16±0.09	0.51±0.10
Identical decisions	88±3% (86)	85±1% (82)	68±2% (68)	70±2% (67)
**Frequency**	62±6%	78±2	84±2%	72±2%
C, σ	5,5	1,1	10,10	5,1
Correlation with bat performance	0.36±0.05	0.61±0.11	0.11±0.18	0.60±0.14
Identical decisions	77±3% (60)	79±4% (72)	70±1% (72)	60±3% (62)

Overall performance of the linear and non-linear SVMs when using either the spectrograms (time and frequency information) or the PSDs (only frequency information) of the calls. The C and σ parameters of the best classifiers are presented. The correlation with the bats' performance is the linear correlation coefficient between the bats' performance and the distances from the hyperplane, \as explained in the Materials and Methods. This is the parameter used to choose the most suitable model. The identical decisions depict the percent of trials in which the model made the same decision as the bat. The percent of identical decisions expected by chance for two classifiers are given in brackets along with the corresponding performance.

### Comparison of the metrics

**Figure 3:**
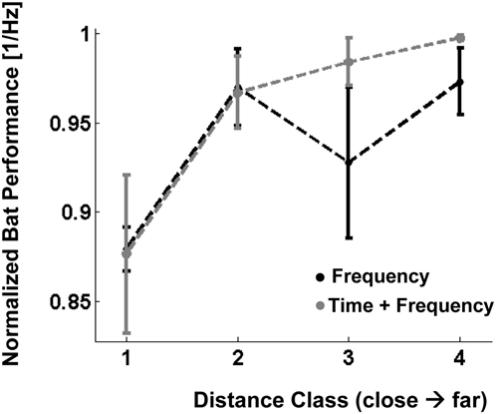
Bats mean performance as a function of the non-linear classifiers' metric – the distance to the hyperplane. The performance of each bat was normalized to a maximum of 1 for the distance class with the highest performance. The distance classes are organized in increasing distances from the hyperplane - i.e., 4 is the class farthest from the hyperplane (easiest to classify), while 1 is the closest (most difficult to classify). The positive correlation implies that the model behaves similarly to the bat.

Our main goal was to model the behavior of the bats. Therefore, more than the overall performance, we were interested to find a classifier that behaves like the bat in the sense that it makes more errors in trials that the model considers to be more difficult and vice versa. We assessed the similarity between the bat and its model by measuring the correlation between the performance of the bat and the performance of the model on the same test set (see Materials and Methods). The performance of the model was indirectly measured by calculating the distances between the pairs of calls in the test set. This reflects the metric of the model. A high correlation between the two indicates that the bat made more errors in trials that are considered to be difficult by the machine and vice versa. Except for a single case (using the PSD for the classification task of bat 6 vs. bat 1) the metrics (distances to the hyperplane) of the linear classifiers are actually negatively correlated with the error rate of the bats, implying that they were using different features than the model to classify the calls (Table 3). We were, however, able to train non-linear SVMs that correlated with the bat's behavior in each of the classification tasks. This was true both for the spectrograms and the PSDs, although the correlation seems a bit less salient in the case of the PSDs (Figure 3). The overall performance of the non-linear SVMs behaving most similarly to the bats was very close to that of the bats, when using the spectrograms and was a bit lower when using the PSDs (Table 3). In one case (classification of bat 5 vs. bat 2) the performance when using the PSDs was much lower.

### Single cue comparisons

To eliminate the possibility that a single simple cue was sufficient for classification we analyzed the commonly used call parameters (starting/terminal/maximum energy frequencies, bandwidth and call duration, Table 1) and tested the performance when relying solely on each of them. We used exactly the same pairs of calls that were presented to the bats in the testing phase and measured the percent of correct decisions if the bat would rely on one of the above parameters, (e.g. always go to the call with a lower or higher terminal frequency). In almost all cases, relying on any single cueresulted in a performance at chance level (45–55%). For the classification task of bat 2 vs. bat 5, using two single cues (the bandwidth or the initial frequency) was sufficient to correctly classify 60–65% of the calls - higher than chance but much lower than the observed performance.

## Discussion

The voice of individual greater mouse-eared bats is specific enough that they can distinguish between the echolocation calls of conspecifics despite their extremely short duration and highly situation-dependent variability. The bats were able to generalize their knowledge to recognize the rewarded individual (S+) and avoide the unrewarded one (S−) when presented with the calls of new individuals that they had not heard during training (S0). A standard linear classifier (SVM) can be trained to fulfill the recognition task with an overall performance similar to that of the bats. The linear models, however, did not reproduce the decision metrics of the bats, implying that the discriminative features they were using were not the ones used by the bats. The linear model can be extended (after a nonlinear transformation of the data with an RBF kernel) to reproduce the behavior of the bats, in other words, the bats made more errors in trials that were considered difficult by the model. Thus, the analysis of these classifiers provides candidate discriminative features derived from the call statistics that might be used by the bats to distinguish between individuals.

**Figure 4:**
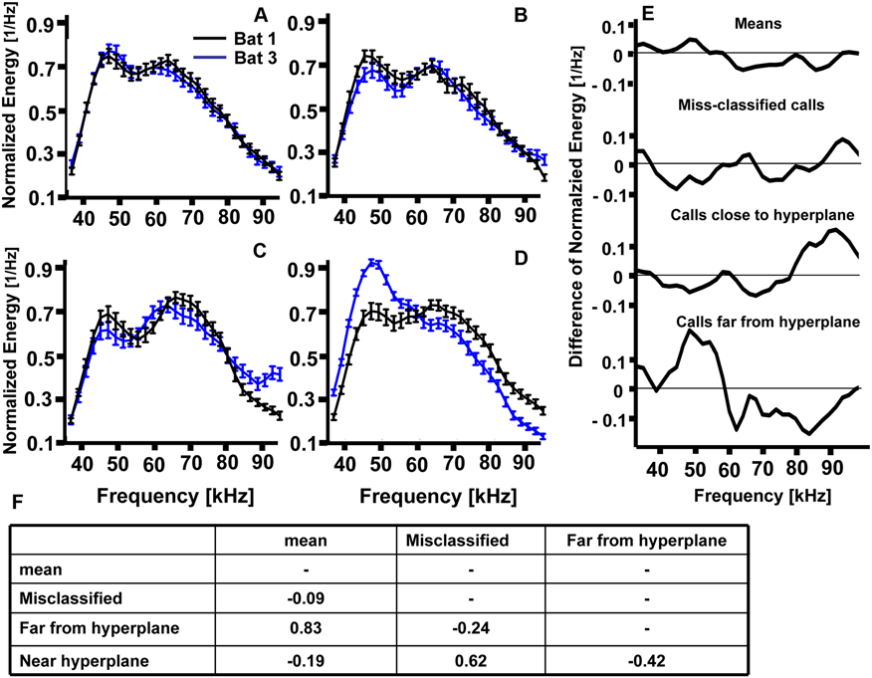
Normalized PSDs (mean and SEM) of the calls of bat 1 (black) and bat 3 (blue). (A) The mean of all calls; (B) The mean of calls misclassified by the bats; (C) The mean of the 15 calls closest to the hyperplane; (D) The mean of the 15 calls farthest from the hyperplane. (E) Difference between the mean PSDs of bat 1 and bat 3 for the four groups of calls shown in A–D. (F) Linear correlation coefficients (a measure of similarity) between the curves presented in Figure 3E representing the difference between the average PSDs (Figure 3A–D).

Examining the PSDs of the calls is a straight-forward approach of searching for spectral individual-specific features. The PSDs of two bats (Figure 4A) reveal a general bimodal pattern in both bats with energy peaks around ∼65 kHz and ∼45 kHz. bat 1 (black), however, tends to have a higher average energy than bat 3 in the 65 kHz peak, while bat 3 (blue) tends to have higher energy in the ∼45 kHz peak.

An extremely over-simplified classification rule could be: “The call with lower energy at ∼65 kHz and higher energy at ∼45 kHz belongs to Bat 3 (S+).” An SVM, however, does not use a single feature, such as the energy at 65 kHz, to classify, but rather takes advantage of all possible cues and their combinations. Examining the PSDs according to the decision rule learned by the SVM can provide some insights about the relative importance of different features (Figure 4). The most obvious observation is that the average difference between the PSDs of calls near the hyperplane is most similar to the average difference between the misclassified calls. This is supported by a high correlation coefficient (0.62, Figure 4F). This means that the calls that are difficult to classify for bats are also difficult for the machine and vice versa. An even more interesting observation is that the average difference between calls far from the hyperplane is very similar to the average difference between all calls, supported by a very high correlation coefficient (0.83). Actually it can be described as an emphasized version of the average difference between all calls.

### Prototype classification

This last similarity implies that the decisions of the bats can be modeled as a prototype classifier [31] in the sense that the bat learns the mean calls of the bat pair as a prototype for the two classes (S+/S−). To test this hypothesis we applied a simple prototype classifier to our data. We used the nearest mean-of class prototype classifier, in which each class is represented by its mean and each call is assigned to the class whose mean PSD is closer to its PSD using the Euclidean distance. The means were calculated from the training data exclusively. Since the bats heard two calls in each trial, we calculated the sum of distances between the PSDs of these calls and the mean PSDs for both the correct and the incorrect assignments. We considered any case for which the correct sum of distances was smaller than the incorrect sum of distances as a correct decision of the classifier. We repeated this for the spectrograms as well.

**Figure 5:**
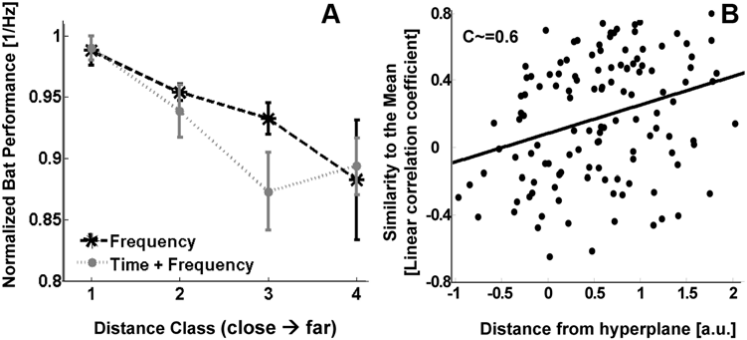
Testing the prototype hypothesis. (A) The mean normalized performance of the bats as a function of the sum of prototype distances. The performance of each bat was normalized to a maximum of 1, for the distance class with the highest performance. The distance used was the sum of Euclidian distances from the pair of calls to the means of the classes. The distance classes are organized according to the distances from the prototype: 4 is the farthest class from the prototype, while 1 is the closest. In contrast to the distances from the SVM hyperplane, for the prototype classifier far means far from the prototype and therefore difficult to classify. We thus expected to find a negative correlation between performance and distance, which is what happened. (B) The similarity between the test call pairs of bat 1 and bat 3 and the mean difference between spectrograms. X axis depicts the distance between the calls according to the SVM metric. The strong positive correlation (linear coefficient C = ∼0.6) implies that the pairs that are more similar to the mean are considered easier to classify by the model.

Despite its simplicity, the prototype classifier achieved a classification performance significantly higher than chance level for both the PSDs and the spectrograms (Table 4). The lower performance compared to the non-linear SVM is not surprising due to the simplicity of this classifier. The overall performance however, is less important in our case. It could probably be increased by a more sophisticated prototype classifier, for instance one that only learns the means of features that have a large inter-bat variability. Much more important is the very high correlation between the distance metric of this classifier (sum of prototype distances) and the bat performance, meaning that the bats tend to make more errors when the calls presented to them are farther from the mean calls (Figure 5A).

**Table 4:** The Performance of a Prototype Classifier.

Classification task\Information used	Bat 2 vs. bat 5	Bat 1 vs. bat 6	Bat 2 vs. bat 6	Bat 1 vs. bat 3
Time+Frequency	70±3%	70±4%	70±5%	64±3%
Correlation with bat performance	−0.65±0.31	−0.32±0.33	−0.27±0.33	−0.67±0.50
Identical decisions	65±3%	62±4%	62±3%	61±3%
Frequency	73±1%	62±3%	69±5%	59±4%
Correlation with bat performance	−0.58±0.12	−0.85±0.13	−0.18±0.33	−0.92±0.10
Identical decisions	68±1%	60±3%	64±3%	52±2%

The performance of a prototype classifier for the different tasks when using the spectrograms (time+frequency information) or PSDs (frequency only). The identical decisions are as in table 3.

An interpretation of the SVM decision rule regarding the spectrograms is not easy due to their high dimensionality, but the above analysis suggests a prototype classifier as well (Figure. 5 and Table 4). To validate this idea we ranked the spectrograms of the presented call pairs of Bat 1 and Bat 3 according to distances between them (based on the non-linear SVM metric). The closer the two spectrograms are to each other, the more difficult they should be to classify. To test the prototype hypothesis we next measured how similar each spectrogram pair is to the pair created by the two class means. We calculated the linear correlation between a) the difference between the pairs and b) the difference between the mean spectrograms. We found a strong positive correlation between the two.which shows that the more similar the difference between two spectrograms is to the mean difference, the easier it is to classify by the trained SVM. As this SVM was trained to imitate the bat's behavior, this once again supports the hypothesis that the bats are using some sort of a prototype classifier (Figure 5B).

In summary, for both PSDs and spectrograms, we found evidence that the bats use a prototype classifier in which they evaluate the mean difference between the calls of the bat couple as a reference to which they compare the difference between any new pair of calls they hear. This hypothesis is strengthened by the results of the generalization experiments, which suggest that the bats are using both S+ and S− to classify (Table 3). We did not observe the exact PSDs of all classification tasks, mainly because the amount of errors for the other tasks was very small. The application of a prototype classifier (Table 4 and Figure 5A) however, implies that all of them were using a sort of a prototype classifier.

### Conclusions

Researchers were always fascinated by the social behaviors exhibited by bats. There are, for instance, some reports of bats leaving the roost and flying to and between foraging sites in groups of between two and six individuals [16],[22]. Little is known about how bats might perform the strenuous task of remaining in a group when flying at high speeds in darkness, or about how they avoid interference between each others' echolocation calls. The finding that bats can recognize their conspecifics based on their echolocation calls might have some significant implications in this context.

Despite their stereotyped spectrograms, echolocation calls show a large task-dependent variability that obscures possible features in the calls that might facilitate the recognition of individual bats [30]. For this reason, we had to use statistical classifiers as a new method of analysis in a context that requires a minimal set of restrictive assumptions on candidate discriminative features. The results pointed strongly towards a prototype strategy. This now enables us to design additional behavioral experiments to test this hypothesis. To test the prototype hypothesis one could, for instance, divide the calls of one of the bats into 2 subgroups that are selected such that their prototype (mean) is very different. The tested bat should then be trained using calls from one subgroup and tested using calls from the other. If the prototype hypothesis holds, the bat would be expected to have a very high error rate. An alternative approach could be to use the hyperplane learnt by the SVM to simulate artificial calls at known distances from the hyperplane and therefore known difficulty [see 32 for more details].

Comparing the performance of the tested classifiers on the PSDs or on the spectrograms reveals that the performance when using the PSDs does not drop as we would expect from taking into account the drop of information (Table 3). This implies that most of the information necessary for classification already exists in the frequency domain. Along with the above analysis of PSDs, this suggests that the filtering properties of the vocal tracts of the individuals, which reflect vocal tract resonances (formants) provide sufficient acoustic cues for individual recognition. These findings are in line with some recent evidence supporting the presence of formants in animal calls [8]–[10], [33]–[35]. It is quite probable that for the classification of the complete repertoire of *M. myotis* calls, including calls emitted in different behavioral situations that show a much higher variation of temporal-spectral relations, the PSDs might even be advantageous compared to the spectrograms since they provide a time-independent set of cues.

## Materials and Methods

### Animals

We conducted the experiments using five adult male *M. myotis* (Borkhausen, 1797), captured in Bulgaria (license from the Ministry of Environment and Waters, 34/04.07.2005, Sofia, Bulgaria) and housed under standardized conditions (16∶8 h light∶ dark cycle, 24±2°C and 65±5% humidity). Bats were fed on mealworms (larvae of *Tenebrio molitor*) only during training and experimental sessions. The diet was supplemented with minerals (Korvimin®, WDT) and vitamins (Nutrical©, Albrecht) and freshwater was accessible all the time. The animals used in the experiments were kept together for a few months in a flight cage that enabled them to fly regularly.

### Data acquisition

Five bats were recorded separately while freely flying in a flight room (3.6×6.0×2.8 m) covered with acoustic foam to reduce echoes from the walls and floor. The flight behavior consisted of two patterns: The animals either circled in the room ca. 2 m above ground, or they flew to one of the walls and hung on it. In the latter case we encouraged them to fly again by clapping the hands or gently poking them with a butterfly net. The sound recordings were performed with custom-made equipment (Universität Tübingen, Germany) including an ultrasonic microphone (flat response ±3 dB between 18 and 200 kHz) in a stationary position pointing 45° upwards at one end of the room and a digital recorder (PCTape), with a sampling rate of 480 kHz. The order of the animals was selected using the Latin squares method [36] to mitigate undesired effects caused by the order or time of the day.

The recordings lasted 20 minutes in total, collected on two consecutive days. This procedure provided us with a large data set of over 2000 calls per bat. The characteristics of the calls varied greatly within each individual even though they were emitted under the same conditions. This variability had at least two causes: 1) Behavioral - the bats were constantly changing their distance from the walls, especially when approaching them to land and adjusted their echolocation accordingly [37],[38]. 2) Acoustical - the calls were recorded when the bats were at different distances from the microphone and with different aspect angles to it. This resulted in substantial changes in the signal to noise ratio (SNR: see Results for more details). We discarded all calls that were shorter than 2 ms since they were severely affected by the directionality of the microphone (i.e. calls with a strong attenuation at high frequencies). This procedure left us with approximately 800 calls for each bat.

### Behavioral classification experiments

In the behavioral experiments each bat was trained to distinguish between two other specific bats in a 2-AFC paradigm. Each experimental bat was assigned two other bats between whose calls it had to distinguish. We will refer to the bat it had to approach as S+ and to the other one as S−. The bats had to sit on a Y-shaped platform and crawl to the side where the calls of S+ were played. The stimuli consisted of alternately playing a single call of S+ on one side of the platform and a single call of S− on the other side with a 0.5 s pause between them until the bat made a decision. All calls were normalized in the time domain to have the same maximum amplitude. We used custom-made equipment (Universität Tübingen, Germany) to play back the calls with a sampling rate of 480 kHz. The loudspeakers (Thiel Diamond Driver D^2^ 20-6) were positioned 1.35 m from the platform and 1.35 m apart from each other, forming an equilateral triangle together with the platform. The side on which S+ was presented varied randomly between the trials. The experiments were divided into a training phase and a testing phase. In the training phase the bats were trained to perform the task using a subset of the data composed of 80% of the calls (the training set) chosen randomly. During training, when the bat crawled to S+, it was rewarded with a mealworm. The bats needed ∼4 days of training to get used to sitting on the Y-platform (they were fed on it). They needed another ∼3 days to learn to crawl to one of the sides of the Y-platform to get the reward. To do this, we placed the bat in the starting arm and played back S+ from one side and S− from the other one, showing the mealworm at the end of the correct arm and rewarding the bat for crawling towards it. The next step (the training phase) consisted of the training on the task. S+ and S− were played back as described above and the bats were rewarded for crawling to the correct side. When they made an error the trial would be repeated up to 3 times. If the bat continued misclassifying we moved to the next pair of calls. Once a bat made more than 75% correct decisions\3 days in a row it was transfered into the testing phase. The training phase lasted ∼20 days on average so that each bat performed ∼25 trials per day so that in total the bats heard ∼500 calls of each bat before starting the testing phase. In the testing phase, we used the remaining 20% of the calls that had never been heard by the bats before. Each pair of calls was played back during a single trial. The decision of the bats was always rewarded, so that the experimenter could not give the bats a hint about the correct answer (a double blind paradigm). The assignment of bat pairs (S+ vs. S−) were as following: bat1–bat2 vs. bat6, bat3–bat6 vs. bat1, bat4–bat5 vs. bat2 and bat5–bat3 vs. bat1. We used four different pair of bats (rather than testing all bats on the same task) assuming that all tasks were more or less equally hard and thus a high performance in all of them would imply high performance for any chosen pair of bats.

### Controls

We recorded the calls that were played back by the speakers to validate that the system was working properly with the same recording equipment mentioned above.

### Test of generality

To test the ability of the bats to generalize and to estimate whether they learned to recognize S+ or to avoid S− we conducted another set of control experiments. Here S+ or S− were presented on one side and S0, which consisted of a call of one of two novel bats never played back to that animal before, on the other side. The S+/S− calls were randomly selected from the training set, since the bats recently heard all of the testing calls and were not exposed to training calls for at least 2 weeks. The order of presentation of S+ or S− and S0 was random as well as the side on which they were played. The rest of the procedure was the same as in the testing session.

### Machine classification

We used Support Vector Machines [Bibr pcbi.1000400-Cristianini1][SVM],[39,40], a well-known classification algorithm in the field of machine learning, to classify the calls of the different bats. This method is suitable for dealing with multi-dimensional data and uses the raw data in order to learn the best features for classification, with minimal prior assumptions on the data distribution.

### Data preprocessing

We tested the performance of the classifier using two different representations of the calls: spectrograms and power spectral densities (PSD). The spectrograms are a time-frequency decomposition of the calls and therefore represent both types of information the bats possess after the basic filtering in the ear [41]. The spectrograms were calculated using a Hann FFT window of 240 points with 0.9 overlap between consecutive windows, providing a frequency resolution of 2 kHz and a time resolution of 0.5 ms. The part of the spectrogram containing the call was segmented from the background noise using Otsu's method [42]. This was done for each spectrogram separately and provided us with the call segments that were clearly above noise. We should emphasize that this was done for the machine classification only. The bats had to face noisy calls with a large variability of background noise.

We restricted the spectrograms to the frequency range between 21–140 kHz, which contains the entire frequency range of the calls. This left us with very high-dimensional data (4200 dimensions: 60 frequencies times 70 time points). We aligned all spectrograms in the time axis such that in all calls the maximal energy at 30 kHz was at the same time instant of the spectrogram. We used Principal Component Analysis (PCA) to reduce the dimensionality of the data. Each data point (representing a single call) was projected on the 300 eigenvectors with the highest eigenvalues. This reduced the dimensionality of the data to 300 dimensions. In a spectrogram of a frequency-modulated *M. myotis* call most of the values of each spectrogram contain background noise. Reducing the dimensionality in a way that preserves the directions of the greatest variance (using PCA) should therefore get rid of a large amount of noise. In every experiment, the eigenvectors were exclusively calculated from the covariance matrix of the training set (see below).

The PSD contains only the frequency information of the calls, leading to a classification that is independent of temporal information (e.g., call duration, sweep rate) which tends to vary widely in nature. Throughout the paper they will sometimes be referred to as spectra. The PSDs were calculated with Welch's method with a 2 ms window with 0.5 overlap. We then under-sampled the PSDs so that their frequency resolution was identical to that of the spectrograms, ensuring that they contained the same spectral information as the spectrograms but no temporal information. All data points (spectrograms after PCA and PSDs) were normalized (divided by the maximum) so that each of them had a maximum of 1 before they were used for classification.

### SVM classification

SVMs are state-of-the-art learning algorithms based on statistical learning theory. A linear SVM uses a training data set to learn a hyperplane (a multidimensional decision boundary) that divides the data set into two classes. It does so by minimizing the classification error and at the same time by maximizing the distance between the hyperplane and the data points that are closest to it. A non-linear SVM is used when the data cannot be separated linearly. It first transforms the data non-linearly into a higher-dimensional space (feature space) and then finds a hyperplane that divides the data into the two classes in this space. In both cases the hyperplane is simply a geometrical multidimensional plane either in the original or in the feature space. Since in many cases a perfect separation of the data into two classes is not possible, the learning algorithm is adjusted to enable a certain amount of misclassification. This is controlled by a constant (C) that defines the penalty for misclassified points. This constant is known as the free parameter of the SVM.

We applied SVM classifiers on both types of data (i.e., spectrograms and PSDs). We used the same training set of calls that was used to train the bats in order to train the classification machines and the same test set to test them. We tested both linear and non-linear SVMs. For the non-linear SVMs, we trained non-linear machines using the radial basis Gaussian kernel [Bibr pcbi.1000400-Cristianini1][RBF],[39],[40,43] to transform the data nonlinearly before computing the separating hyperplane. This is a standard choice in machine learning that usually performs well in a wide range of applications. The use of the RBF kernel introduces a second parameter (σ) that sets the width of the Gaussian. In order to optimize the classifier to perform like the bat (see below) we tested 8 different values for each of the two parameters (0.1, 1, 10, 50, 100, 500, 1000, 10000) and trained linear SVMs with all possible C values and non-linear SVMs with all possible combinations of the two in order to find a classifier with a performance that is most similar to that of the bats.

### Model selection

There are several possibilities to optimize the model such that it behaves like a bat. The overall performance (error rate) is not a sufficient criterion since it does not provide any information about the classification strategy - e.g., the bat and model could do the exact opposite right and wrong decisions but still have the same error rate. An exact comparison between the decisions of the bat and the decisions of the model (percent of identical right/wrong decisions) is a better criterion, but it is also limited since it divides the trials into identical decisions and non-identical decisions but provides no information about how difficult each decision was. We therefore chose a different criterion, one which is, to our understanding, more informative. For each model (linear/non-linear SVM) we computed the distances between the pairs of test calls the bat had to classify according to the model. This can be done by computing the distance of each call from the hyperplane. The distance from the hyperplane can be thought of as an estimation of how difficult the call is to classify. The closer a call is to the hyperplane, the more difficult it is to classify, since it is closer to the boundary between the two classes. We refer to this measure as the metric of the model and it reflects how difficult/easy each trial is considered to be according to the model. We assumed that if the machine captured the features used by the bats for classification, the distance between the calls should positively correlate with the performance of the bats, meaning that the farther apart the two calls presented to the bat were, the easier it should be for the bats to classify them correctly. In practice we divided the entire distance range into 4 distance classes, each containing an equal number of calls and plotted the error rate of the bats for each of these distance ranges. We then calculated the correlation between the performance of the bat and the difficulty of the trials it performed, represented by the average distances of the group of trials. We searched for the parameters that yielded a classifier that maximizes this correlation. To choose the best parameters we divided the test set into 3 equally sized sub-sets of data. We then used only two thirds of the test set to choose the best model (this set is called the validation set) and we measured the results on the un-used third. This process was repeated three times and ensures that the test set did not influence our decision. This procedure also provided us with an estimation of the variance of the model's performance.

We implemented the SVM classifier using the free “spider” software (http://www.kyb.mpg.de/bs/people/spider). For more details about the application of SVMs on a data set of spectrograms see Yovel et al [31].
